# Is platelet transfusion associated with hospital-acquired infections in critically ill patients?

**DOI:** 10.1186/s13054-016-1593-x

**Published:** 2017-01-06

**Authors:** Cécile Aubron, Andrew W. Flint, Michael Bailey, David Pilcher, Allen C. Cheng, Colin Hegarty, Antony Martinelli, Michael C. Reade, Rinaldo Bellomo, Zoe McQuilten

**Affiliations:** 1 Australian and New Zealand Intensive Care Research Centre, Department of Epidemiology and Preventive Medicine, Monash University, Melbourne, Australia; 2 Transfusion Research Unit, Department of Epidemiology and Preventive Medicine, Monash University, Melbourne, Australia; 3 Intensive Care Unit, The Alfred Hospital, Melbourne, Australia; 4 Intensive Care Unit, Austin Hospital, Melbourne, Australia; 5 Department of Infectious Disease, The Alfred Hospital, Melbourne, Australia; 6 Department of Epidemiology and Preventive Medicine, Monash University, Melbourne, Australia; 7 Burns Trauma and Critical Care Research Centre, University of Queensland, Herston, Queensland 4029 Australia; 8 Transfusion Service, Austin Hospital, Studley Road, Heidelberg, Melbourne, Victoria 3084 Australia; 9 Joint Health Command, Australian Defence Force, Canberra, Australian Capital Territory 2160 Australia; 10 Réanimation Médicale, Centre Hospitalier et Universitaire de Brest site La Cavale Blanche - Université de Bretagne Occidentale, Bvd Tanguy Prigent, 29609 Brest Cedex, France

**Keywords:** Platelet transfusion, Hospital-acquired infection, Bacteraemia, Critically ill patients

## Abstract

**Background:**

Platelets are commonly transfused to critically ill patients. Reports suggest an association between platelet transfusion and infection. However, there is no large study to have determined whether platelet transfusion in critically ill patients is associated with hospital-acquired infection.

**Methods:**

We conducted a multi-centre study using prospectively maintained databases of two large academic intensive care units (ICUs) in Australia. Characteristics of patients who received platelets in ICUs between 2008 and 2014 were compared to those of patients who did not receive platelets. Association between platelet administration and infection (bacteraemia and/or bacteriuria) was modelled using multiple logistic regression and Cox regression, with blood components as time-varying covariates. A propensity covariate adjustment was also performed to verify results.

**Results:**

Of the 18,965 patients included, 2250 (11.9%) received platelets in ICU with a median number of 1 platelet unit (IQR 1–3) administered. Patients who received platelets were more severely ill at ICU admission (mean Acute Physiology and Chronic Health Evaluation III score 65 (SD 29) vs 52 (SD 25), *p* < 0.01) and had more comorbidities (31% vs 19%, *p* < 0.01) than patients without platelet transfusion. Invasive mechanical ventilation (87% vs 57%, *p* < 0.01) and renal replacement therapy (20% vs 4%, *p* < 0.01) were more frequently administered in patients receiving platelets than in patients without platelets. On univariate analysis, platelet transfusion was associated with hospital-acquired infection in the ICU (7.7% vs 1.4%, *p* < 0.01). After adjusting for confounders, including other blood components administered, patient severity, centre, year, and diagnosis category, platelet transfusions were independently associated with infection (adjusted OR 2.56 95% CI 1.98–3.31, *p* < 0.001). This association was also found in survival analysis with blood components as time-varying covariates (adjusted HR 1.85, 95% CI 1.41–2.41, *p* < 0.001) and when only bacteraemia was considered (adjusted OR 3.30, 95% CI 2.30–4.74, *p* <0.001). Platelet transfusions remained associated with infection after propensity covariate adjustment.

**Conclusions:**

After adjustment for confounders, including patient severity and other blood components, platelet transfusion was independently associated with ICU-acquired infection. Further research aiming to better understand this association and to prevent this complication is warranted.

## Background

Platelet (PLT) transfusion is used therapeutically in patients who are bleeding [1], and is also recommended by guidelines [2] to reduce the risk of bleeding in patients with thrombocytopenia (prophylactic transfusion). However, the benefit of prophylactic PLT administration in non-bleeding critically ill patients with thrombocytopenia has been questioned [3, 4]. This is important, as there is some evidence suggesting that PLT administration is associated with adverse effects including infection and sepsis [5, 6]. The association between blood component administration and hospital-acquired infection has been reported mainly for red blood cells (RBC) [7–9]. Nonetheless, this association is probably not restricted to RBC, as fresh frozen plasma (FFP) and PLT transfusion can also cause post transfusion immunomodulation [10].

Platelets play a key role in the inflammatory and immune response, [11] and some authors have hypothesised that PLT transfusion may lead to immunomodulation [10, 12]. Nonetheless, there has been no large study of PLT transfusion in ICU patients to investigate whether PLT administration is associated with an increased risk of developing hospital-acquired infection. Patients admitted to an ICU are likely to be particularly prone to transfusion-mediated immunomodulation [13]. Therefore, we aimed to describe PLT transfusion in a large cohort of critically ill patients and to determine whether PLT transfusion is associated with hospital-acquired infection.

## Methods

### Patients and study design

This is a retrospective study conducted in the mixed medical-surgical ICUs of two teaching hospitals in Melbourne (Australia). All patients admitted to one of these ICUs between July 2008 and September 2013 were included in the study. The Alfred Hospital (affiliated to Monash University), with a 45-bed ICU capacity, is a tertiary hospital and state-wide referral centre for trauma and heart and lung transplantation; the Austin Hospital (affiliated to the University of Melbourne), with a 20-bed ICU capacity, is a tertiary hospital and referral centre for liver transplantation for the states of Victoria, Tasmania and South Australia. The Research Ethics Committees of both institutions approved the study (LNR13/Austin/233 and Alfred 58/11).

### Clinical data

Clinical data were those prospectively extracted from local ICU databases used to collect and submit data to the Australian New Zealand Intensive Care Society (ANZICS) Adult Patient Database (APD). Data were collected on gender, date of birth, comorbidities, ICU and hospital admission and discharge dates, Acute Physiology And Chronic Health Evaluation (APACHE III) score at admission, ICU admission categories, requirement for and dates of initiation and cessation of mechanical ventilation (MV) or renal replacement therapy (RRT), and patient vital status at ICU and hospital discharge.

### Transfusion data

Data on all blood components issued and transfused to patients were retrieved from the blood bank laboratory information systems of each hospital. For each PLT unit administered, we recorded the date of transfusion, the type of product (apheresis or pooled), the volume, and the ABO and Rhesus D blood group. Administration of RBC, FFP and cryoprecipitate (CRYO) were also collected. Universal pre-storage leukodepletion was introduced in Australia prior to the study period, and therefore all blood products were pre-storage leukodepleted. Systematic screening of PLT for bacterial contamination was implemented at the same time by the Australian Red Cross Blood Service. PLT transfusion practices in critically ill patients were according to clinician discretion.

The national guidelines in place at the time of the study were the Australian Society of Blood Transfusion (ASBT) guidelines, which recommended prophylactic PLT transfusion at a threshold of 10 to 20 × 10^9^/L in bone marrow failure and a threshold of 50 × 10^9^/L prior to surgery or invasive procedures, [14] and therapeutic PLT transfusion in bleeding patients in whom thrombocytopenia was considered a major contributor factor, when the platelet count was less than 50 × 109/L in the context of massive haemorrhage, or less than 100 × 10^9^/L in the presence of diffuse microvascular bleeding [14]. An observational study conducted in Australia and New Zealand confirmed the compliance with these guidelines in ICU patients during the same study period with only 5% of PLT transfusions that were inappropriate [15]. The updated guidelines published towards the end of the study period specifically for critical care included similar proposed thresholds for prophylactic PLT transfusion in the absence of bleeding (<20 × 10^9^/L) and prior to invasive procedures (<50 × 10^9^/L) [16].

### Microbiological data

Microbiological data were retrieved from the microbiology laboratory records (KESTRAL system, www.kestral.com.au, Melbourne, Victoria, Australia) at the Austin hospital, and from a prospectively maintained database by the infectious disease department at the Alfred hospital. Microbiological data included positive blood culture and positive urine culture (defined by the presence of no more than two bacterial or Candida pathogens, and at 10^5^ colony forming units (cfu)/mL or higher [17]). Sample date and pathogens isolated were recorded. When a pathogen known to be a frequent contaminant of microbiological cultures (i.e. coagulase-negative staphylococcus) was isolated, only cases with two or more positive blood cultures with the same pathogen were considered as bloodstream infection.

### Statistical analysis

Descriptive statistics are reported as mean (standard deviation) or median (interquartile range) according to data distribution. Hypothesis testing was performed using the chi-square test for categorical variables, Student *t* test for normally distributed data and Wilcoxon rank-sum test for non-normally distributed data. The relationship between PLT transfusion and ICU-acquired infection was determined by logistic regression with results reported using odds ratios (ORs) (95% confidence interval (CI)). Model discrimination and calibration were determined using the area under the receiver operating characteristic (ROC) curve and the Hosmer-Lemeshow statistic.

Infection was defined as either bacteraemia or bacteriuria occurring in the ICU after 48 hours of ICU stay. Multivariate analysis was performed adjusting for gender, APACHE III score, site, diagnosis category, year, whether the patient received invasive MV or RRT, and whether another type of transfusion (RBC, CRYO, or FFP) had been given prior to the first dose of PLT. Additional sensitivity analysis was performed, adjusting for each patient’s propensity to receive a PLT transfusion; the propensity variable was calculated by using the predicted values of a multiple logistic regression model of platelet transfusion from pre-admission variables including comorbidity variables and the same variables as the multivariate analysis. Cox proportional hazards regression analysis was then performed, with the outcome of time of first infection, and including transfusion variables as time-varying covariates, and gender, APACHE III score, site, diagnosis category, year, and requirement for RRT and MV. Finally, three additional models were then also performed, considering only bacteraemia or bacteriuria as the infectious outcome and considering the number of platelet units transfused as a continuous variable. Propensity assumptions were determined using Schoenfeld residuals. To increase the robustness of our findings, a two-sided *p* value of 0.01 was considered to indicate significance. Statistical analysis was performed using STATA version 12.1 (StataCorp, TX, USA).

## Results

### Patient characteristics

A total of 19,101 patients had at least one admission to of the either ICUs over the five and half year study period. Of these, 136 (0.7%) were excluded because of missing data, leaving 18,965 included in the present study. Of the 18,965 patients, 2250 (11.9%) received PLT in the ICU.

Patients’ characteristics and comparisons between patients who received PLT in the ICU and those who did not are displayed in Table 1. Patients who received PLT were younger (59 ± 17 vs 60 ± 17, *p* = 0.036) and more often male (67.2% vs 63.3%, *p* < 0.01) than those who did not receive PLT. They had greater illness severity at ICU admission, with an APACHE III score of 65 ± 29 vs 52 ± 25 (*p* < 0.01) and they more often had comorbidities (31% vs 19%, *p* < 0.01). Patients who received PLT in the ICU were more often admitted with cardiovascular disease, gastrointestinal (GI) disease, trauma, or haematological disease than those who did not receive PLT. The majority of patients receiving PLT required invasive MV (87% vs 57%, *p* < 0.01) and one fifth had RRT while in the ICU (20% vs 4%, *p* < 0.01).

**Table 1:** Characteristics of patients with and without platelet (PLT) transfusion in the ICU

Variables	All patients *N* = 18,965	Patients with PLT transfusion *N* = 2250	Patients without PLT transfusion *N* = 16,715	*P* value
Age, years, mean (SD)	60 (18)	59 (17)	60 (17)	0.04
Gender, male, *n* (%)	12 086 (63.7%)	1 511 (67.2%)	10 575 (63.3%)	<0.01
APACHE III score, mean (SD)	54 (26)	65 (29)	52 (25)	<0.01
*Comorbidities*				
Any known comorbidity	3 838 (20%)	702 (31%)	3 136 (19%)	<0.01
Cancer^a^	857 (4.5%)	192 (8.5%)	665 (4.0%)	<0.01
Hepatic disease^b^	674 (3.6%)	216 (9.6%)	458 (2.7%)	<0.01
Immunocompromised^c^	1 180 (6.2%)	311 (13.8%)	869 (5.2%)	<0.01
IDDM	511 (2.7%)	60 (2.7%)	451 (2.7%)	0.93
Chronic respiratory disease	840 (4.4%)	92 (4.1%)	748 (4.5%)	0.40
CVD	754 (4%)	137 (6.1%)	617 (3.7%)	<0.01
Chronic renal failure	422 (2%)	59 (2.6%)	363 (2.2%)	0.17
Admission diagnosis				
Cardiovascular	5 161 (27%)	701 (31%)	4 460 (27%)	<0.01
Gastrointestinal	3 595 (19%)	581 (26%)	3 014 (18%)	<0.01
Haematological	152 (0.8%)	96 (4.3%)	56 (0.3%)	<0.01
Neurological	1 586 (8.4%)	108 (4.8%)	1 478 (8.8%)	<0.01
Renal/genitourinary	1 017 (5.4%)	108 (4.8%)	909 (5.4%)	0.2
Respiratory	1 467 (7.7%)	127 (5.6%)	1 340 (8%)	<0.01
Sepsis	503 (2.7%)	73 (3.2%)	430 (2.6%)	0.06
Other^d^	2 815 (14.8%)	82 (3.6%)	2 733 (16.4%)	<0.01
Trauma	2 669 (14.1%)	374 (16.6%)	2 295 (13.7%)	<0.01
Requirement for MV	11 531 (61%)	1 952 (87%)	9 579 (57%)	<0.01
Requirement for RRT	1 135 (6%)	449 (20%)	686 (4%)	<0.01

^a^Cancer group includes myeloma, lymphoma, leukaemia and metastases. ^b^Hepatic disease group includes liver cirrhosis, chronic liver disease and hepatic failure. ^c^Immunocompromised includes those listed as immunosuppressed or with immune disease. ^d^Other includes undefined, metabolic, muscle and skin, gynaecologic diseases. *PLT* platelets, *ICU* intensive care unit, *MV* mechanical ventilation, *RRT* replacement renal therapy, *APACHE* Acute Physiology And Chronic Health Evaluation, *IDDM* insulin-dependent diabetes mellitus, *CVD* chronic vascular diseases, *SD* standard deviations. *P* values comparing PLT transfusion group to the no-PLT transfusion group

#### Transfusion characteristics

PLT units (n = 6012) were transfused over the study period, with a median of 1 PLT unit (IQR 1–3) per patient. Patients with haematology admission diagnosis received more PLTs than the other critically ill patients. The ABO group of the PLT unit transfused was known in 2685 units and was mostly groups O (56%) and A (40%). PLT transfusions were Rhesus-D-positive in 71% of patients. Patients transfused with PLT were also transfused with RBC, FFP and CRYO in 79%, 62% and 33% of cases, respectively. These figures were significantly higher (*p* < 0.01) than in patients without PLT transfusion (RBC 21%, FFP 6%, cryoprecipitate 0.8%). The first PLT transfusion occurred in the first 24 hours after ICU admission in 82.6% of patients.

#### PLT transfusion and infections

Overall, 411 patients (2.2%) experienced an ICU-acquired infection in ICU that was either bacteraemia or bacteriuria (Table 2). A much higher proportion of patients who had received platelets developed infection (7.7%) compared to those who had not (1.4%) (*p* < 0.01). PLT transfusion was associated with occurrence of both bacteraemia and bacteriuria (Table 2). Patterns of pathogens isolated in blood culture and urine culture are displayed in Tables 3 and 4.

**Table 2:** Platelet exposure and outcomes

Variables	All patients *N* = 18,965	Patients with PLT transfusion *N* = 2250	Patients without PLT transfusion *N* = 16,715	*P* value*
Infections, *n* (%) Any (blood or urine culture) Blood culture Urine culture	411 (2.2) 178 (0.9) 262 (1.4)	173 (7.7) 99 (4.4) 89 (4)	238 (1.4) 79 (0.5) 173 (1)	<0.01 < 0.01 < 0.01
Time to infection, days median (IQR) Any (blood or urine) Blood culture Urine culture	7.1 (4.2–12) 7.9 (4.5–12) 6.8 (4.1–11.7)	8.9 (5.1–15.0) 8.4 (5–14) 9.1 (5.2–17)	6.4 (3.9–10) 6.6 (4.3–12) 6.3 (3.8–10.1)	<0.01 < 0.01 0.25

*PLT* platelets, *IQR* interquartile range. * *P* values comparing PLT transfusion group to the no-PLT transfusion group

**Table 3:** Microbiological features of positive urine cultures occurring in patients with and without PLT transfusion in the intensive care unit

Pathogens	Patients with positive UC *N* = 292	Patients with positive UC and with PLT transfusion *N* = 99	Patients with positive UC without PLT transfusion *N* = 193	*P* value
Enterobactericeae *Enterococcus*	17 (5.8%)	43 (43.4%)	108 (56%)	0.04
*Staphylococcus* sp.	38 (19.5%)	2 (2%)	15 (7.8%)	0.04
*Candida* sp.	7 (2.4%)	3 (3%)	4 (2.1%)	0.44
Non-fermentive GNB	90 (30.8%)	44 (44.4%)	46 (23.8%)	< 0.01
Other	30 (10.3%)	9 (9.1%)	21 (10.9%)	0.63
	2 (0.7%)	1 (1%)	1 (0.5%)	0.56
UC with >1 microorganism	25 (8.6%)	5 (5.1%)	20 (10.4%)	0.12

*PLT* platelets, *GNB* Gram-negative Bacilli; *UC* urine culture. *P* values comparing PLT transfusion group to the no-PLT transfusion group

**Table 4:** Microbiological features of positive blood cultures in patients with and without PLT transfusion

Pathogens	Patients with positive blood culture *N* = 195	Patients with positive blood culture who had PLT transfusion *N* = 108	Patients with positive blood culture without PLT transfusion *N* = 87	*P* value
Enterobactericeae	54 (27.7%)	28 (26%)	26 (29%)	0.54
*Enterococcus*	38 (19.5%)	27 (25%)	11 (13%)	0.03
*Staphylococcus aureus*	20 (10.3%)	9 (8.3%)	11 (12.6%)	<0.01
CNS	39 (20%)	21 (19.4%)	18 (20.7%)	0.83
Candida	36 (18.5%)	26 (24.1%)	10 (11.5%)	0.02
Non-fermentive GNB	16 (8.2%)	7 (6.5%)	9 (10.3%)	0.33
Other	2 (1%)	1 (0.9%)	1 (1.2%)	0.88
BC with >1 microorganism	14 (7.2%)	9 (8.3%)	5 (5.8%)	0.49

*PLT* Platelets, *CNS* coagulase negative Staphylococcus, *GNB* Gram-negative Bacilli, *BC* blood culture. *P* values comparing PLT transfusion group to the no-PLT transfusion group

After adjusting for confounders (gender, APACHE III score, site, diagnosis category, year, whether another type of transfusion had been given prior to the PLT transfusion, MV and/or RRT requirement), PLT transfusion was associated with an increased risk of infection (adjusted odds ratio (OR) 2.56, 95% confidence interval (CI) 1.98–3.31, *p* < 0.01). The other independent risk factors for infection were female gender, patient severity at admission, requirement for invasive MV, requirement for RRT, admission diagnosis, administration of RBC prior to PLT transfusion and year (Table 5). As there were no significant interactions between PLT transfusion and other variables in the model, there was no evidence to suggest that the relationship between PLT transfusion and infection differed according to any of the covariates considered, including diagnosis category. When adjusting for the propensity to receive platelet transfusion, PLT transfusion remained associated with an increased risk of infection (Appendix).

**Table 5:** Independent risk factors for infection in a multivariate analysis

Variables^a^	Adjusted odds ratio*	95% Confidence interval	*P* value
PLT	2.56	1.98-3.31	<0.01
Gender, male	0.40	0.33-0.50	<0.01
APACHE III score	1.01	1.00-1.01	<0.01
RBC transfusion^b^	1.50	1.19-1.90	<0.01
FFP transfusion^b^	0.92	0.68-1.24	0.57
Cryoprecipitate^b^	1.14	0.75-1.73	0.53
Invasive mechanical ventilation	3.16	2.29-4.36	<0.01
RRT	4.75	3.56-6.32	<0.01

^a^Other confounders include centre, year and admission diagnosis. ^b^Only blood products transfused prior to platelets (PLT) transfusion are considered in the analysis. *RBC* red blood cells, *FFP* fresh frozen plasma, *RRT* renal replacement therapy, *APACHE III* Acute Physiology And Chronic Health Evaluation III. *Hosmer-Lemeshow *p* value >0.54, area under the receiver operating characteristic curve 0.83

**Fig. 1:**
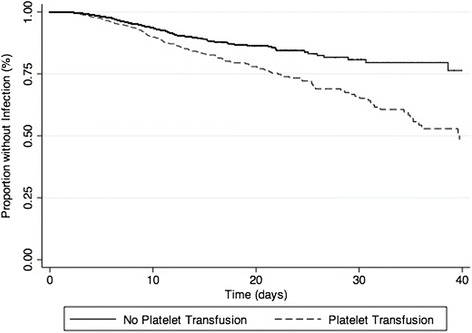
Kaplan-Meier estimates of infection in all patients over 40 days after ICU admission (*p* < 0.01 by the log-rank test)

**Fig. 2:**
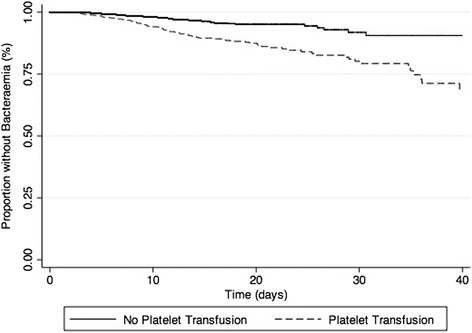
Kaplan-Meier estimates of bloodstream infections in all patients over 40 days after ICU admission (*p* < 0.01 by the log-rank test) )

When considering blood product transfusion (PLT, RBC, FFP or CRYO) as time-varying covariates, PLT administration remained associated with infection (adjusted HR 1.85, 95% CI 1.41–2.41, *p* < 0.01) (Table 6). When considering bacteraemia and bacteriuria separately, PLT transfusion was still associated with each infection outcome (adjusted OR for bacteraemia 3.30, 95% CI 2.30 − 4.74, *p* <0.01 and adjusted OR for bacteriuria 2.01, 95% CI 1.44–2.83, *p* <0.01). There was a dose effect in the association between PLT transfusion and infection (adjusted OR for one PLT unit 1.62, 95% CI 1.11–2.35, *p* = 0.01; adjusted OR for two PLT units 3.48, 95% CI 2.60–4.68, *p* < 0.01). Figure 1 shows the survival time analysis for infection in patients with and without PLT and Fig. 2 shows the survival time analysis for bacteraemia in patients with and without PLT.

**Table 6:** Independent risk factors for infection in the Cox model

Variables^a^	Adjusted hazard ratio	95% Confidence interval	*P* value
PLT transfusion	1.85	1.41-2.41	<0.01
Gender	0.43	0.35-0.52	<0.01
APACHE III score	1.00	1.00-1.01	0.20
RBC^b^	1.02	0.79-1.33	0.86
FFP^b^	0.87	0.66-1.14	0.32
Cryoprecipitate^b^	1.01	0.74-1.40	0.94
Invasive mechanical ventilation	0.77	0.57-1.06	0.11
RRT	1.33	1.03-1.71	0.03

^a^Other confounders include centre, year and admission diagnosis. ^b^Only blood products transfused prior to infection are considered in the analysis. *RBC* red blood cells, *FFP* fresh frozen plasma, *RRT* renal replacement therapy, *PLT* platelets, *APACHE* Acute Physiology And Chronic Health Evaluation

## Discussion

In this large observational study, PLT transfusion was associated with ICU-acquired infection. This association remained after adjustment for important confounders, including administration of other blood products prior to PLT administration and when considering all blood components as time varying covariates. PLT transfusion was still an independent risk factor for infection when considering separately bacteraemia and bacteriuria as an outcome.

### Comparison with the available literature

It has been suggested that there is an association between infections and PLT administration in trauma and post-cardiac-surgery settings [5, 6, 18]. In a post hoc analysis of data from six randomised trials, Spiess et al. found that PLT transfusion was associated with an increased risk of critical adverse events including infection [5]. Nonetheless, the study conducted by Spiess et al. was performed before the widespread use of leukoreduction of blood components and its statistical analysis did not adjust for all other blood components administered.

More recently, Bilgin et al. reported on 1085 cardiac surgery patients in whom the risk of death due to post-surgical infection was independently associated with PLT transfusion [6]. However, the criteria to define infection were not objective, with infection diagnosis based on the “physician’s opinion”. In both studies, microbiology data were not provided. In contrast, there was no association between PLT transfusion and infection or morbidity in other large cohorts of patients who had undergone cardiac surgery [19–21]. Despite these conflicting results, the plausibility of this association is supported by reports showing the independent role of PLT in transfusion-related immunomodulation in animals [10].

### Strengths and limitations

To our knowledge, this is the largest available study investigating the association between PLT transfusion and hospital-acquired infections in critically ill patients. Its heterogeneous population and its multi-centre design support the generalizability of our findings. The consistent results with different modelling approaches and the consideration of transfusion of blood components as time-varying to overcome survival bias is another strength that has not been used commonly in similar studies on this topic. The use of prospectively maintained databases of infectious outcomes and microbiology criteria to define infection also reduced bias, which may be introduced if relying on subjective definitions of infection by clinicians.

Nonetheless, our study has some limitations. Its retrospective design did not allow adjustment for relevant but missing data. Although we did perform propensity covariate adjustment to account for the probability of receiving a PLT transfusion, estimation of the propensity score could only be based on the available data. Infections that could not be reliably identified in our data could not be included in the analysis, such as ventilator-associated pneumonia or abdominal infections. Bacteraemia sources were not recorded, making it impossible to classify bacteraemia according to the infection source. There was no information on whether the patient was on antibiotics at the time the microbiology samples were obtained. Such information would have been useful to better understand the relatively low rate of infection in our population. Data were not available on blood components given prior to ICU admission, and infections occurring after ICU discharge. Finally, although we adjusted for important factors known to influence the risk of infection, we cannot exclude the possibility that the observed associations were confounded by unmeasured factors.

### Implications of study findings

Our results suggest an association between PLT transfusion and adverse clinical outcomes, and emphasise the importance of avoiding unnecessary transfusions. Avoiding unnecessary transfusion should be a priority and may decrease hospital-acquired infection [3].

## Conclusion

In conclusion, we found an independent association between PLT transfusion and the risk of hospital-acquired infections in critically ill patients. This association should be taken into account when transfusing PLT in critically ill patients. Further research to understand this association and to better determine the benefit of PLT in this population is warranted.

## Appendix

### Propensity covariate adjustment

**Table 7:** Independent risk factors for infection in a multivariate analysis including propensity covariate adjustment

Variables^a^	Adjusted odds ratio	95% Confidence interval	*P* value
PLT	2.45	1.89-3.18	<0.01
Gender	0.40	0.32-0.49	<0.01
APACHE III score	1.00	1.01-1.01	0.01
RBC transfusion^b^	1.39	1.08-1.78	0.01
FFP transfusion^b^	0.69	0.46-1.02	0.06
Cryoprecipitate^b^	0.70	0.39-1.28	0.25
Invasive mechanical ventilation	2.89	2.07-5.46	<0.01
RRT	3.90	2.79-5.46	<0.01
Propensity covariate^c^	3.46	1.15-10.38	0.03

^a^Other confounders include centre, year and admission diagnosis. ^b^Only blood products transfused prior to platelets (PLT) transfusion are considered in the analysis. ^c^Propensity covariate predicted by gender, site, diagnosis, immune disease, immunosuppressed, chronic liver disease, year, red blood cells (RBC) transfusion, fresh frozen plasma (FFP) transfusion, cryoprecipitate transfusion, invasive mechanical ventilation, renal replacement therapy (RRT), age, leukaemia/myeloma, metastases, hepatic failure, insulin-dependent diabetes. *APACHE* Acute Physiology And Chronic Health Evaluation

## Abbreviations

APACHE: Acute Physiology And Chronic Health Evaluation
CI: Confidence interval
Cryo: Cryoprecipitate
FFP: Fresh frozen plasma
HR: Hazard ratio
ICU: Intensive care unit
IQR: Interquartile range
MV: Mechanical ventilation
OR: Odds ratio
PLT: Platelets
RBC: Red blood cells
RRT: Renal replacement therapy
